# A Simple Fitness Function for Minimum Attribute Reduction

**DOI:** 10.1155/2015/921487

**Published:** 2015-08-03

**Authors:** Yuebin Su, Jin Guo, Zejun Li

**Affiliations:** ^1^School of Information Science and Technology, Southwest Jiaotong University, Chengdu 610031, China; ^2^School of Science, Sichuan University of Science and Engineering, Zigong 643000, China

## Abstract

The goal of minimal attribute reduction is to find the minimal subset *R* of the condition attribute set *C* such that *R* has the same classification quality as *C*. This problem is well known to be NP-hard. When only one minimal attribute reduction is required, it was transformed into a nonlinearly constrained combinatorial optimization problem over a Boolean space and some heuristic search approaches were used. In this case, the fitness function is one of the keys of this problem. It required that the fitness function must satisfy the equivalence between the optimal solution and the minimal attribute reduction. Unfortunately, the existing fitness functions either do not meet the equivalence, or are too complicated. In this paper, a simple and better fitness function based on positive domain was given. Theoretical proof shows that the optimal solution is equivalent to minimal attribute reduction. Experimental results show that the proposed fitness function is better than the existing fitness function for each algorithm in test.

## 1. Introduction

For a given dataset, attribute reduction is a fundamental problem in rough set theory as proposed by Pawlak and Sowinski [1]. Formally, it is a nonlinearly constrained combinatorial optimization problem whose objective function is the size of a candidate subset of attributes and whose constraints, represented in terms of classification quality of attribute subsets, are the conditions met by an attribute reduction. As shown by [2], this problem has been proven to be NP-hard.

To overcome this problem, many strategies had to be considered in the literatures during the past two decades. In general, there are two kinds of categories for minimum attribute reduction: greedy (or hill-climbing) categories and stochastic categories. The greedy categories usually employ rough set attribute significance as heuristic knowledge. It starts off with an empty set or attribute core and then adopts forward selection or backward elimination. Hu and Cercone give a reduction algorithm using the positive region-based attribute significance as the guiding heuristic [3]. Wang et al. develop a conditional information entropy-based reduction algorithm, using conditional entropy-based attribute significance [4]. Hu et al. compute the significance of an attribute making use of heuristic ideas from discernibility matrices and propose a heuristic reduction algorithm [5]. Susmaga considers both indiscernibility and discernibility relations in attribute reduction [6]. These categories are fast but do not guarantee to find an optimal or minimal reduction.

Some researchers use stochastic methods for rough set attribute reduction. These categories are optimization methods. It has a higher probability of finding a minimum reduct than the first category. Wroblewski combines a genetic algorithm with a greedy algorithm to generate short reducts. However, it uses highly time-consuming operations and cannot assure that the resulting subset is really a reduct [7]. Taking Wroblewski's work as a foundation, Bjorvand and Komorowski apply genetic algorithms to compute approximate reducts [8]. The algorithm makes several variations and practical improvements both in speed and in the quality of approximation. The reduct generation algorithms based on genetic algorithms for the rough set attribute reduction are quite efficient [9].

But rough set can only deal with the discrete attributes. A method for discretization based on particle swarm optimization (PSO) is presented in [10]. Taking this work as a foundation, an algorithm for knowledge reduction in rough sets is proposed based on particle swarm optimization in [11]. This algorithm can solve some problems that the existing heuristic algorithm cannot solve. In order to improve the efficiency of the algorithm, many scholars constantly improve and update these algorithms. Santana-Quintero Luis et al. present a multiobjective evolutionary algorithm which consists of a hybrid between a particle swarm optimization approach and some concepts from rough sets theory [12]. The main idea of the approach is to combine the high convergence rate of the particle swarm optimization algorithm with a local search approach based on rough sets which is able to spread the nondominated solutions found. After that, two-step particle swarm optimization to solve the feature selection problem was given by Bello et al. in [13]. The improved algorithm is a method which can improve the search efficiency. Chi et al. presented a method for continuous attribute discretization based on quantum PSO algorithm [14]. Hsieh and Horng presented a method for feature selection based on asynchronous discrete PSO search algorithm [15]. Also, other stochastic algorithms were used to attribute reduction, for example, ant colony algorithm (ACO) [16] and support vector machine (SVR) [17].

For systems where the optimal or minimal subset is required, stochastic category may be used. In this case, this problem is transformed into a problem of finding a maximum (or minimum) value of a fitness function at first, and then some stochastic optimization method is applied to solve the fitness maximization (or minimization) problem. A common way to transform a constrained optimization problem into an unconstrained fitness optimization problem is to use penalty methods [18]. For such methods, designing a better fitness function is the most important work. To get good performance, the fitness functions should meet the requirements that the fitness evaluation of a candidate solution is appropriate and the optimality equivalence is guaranteed. Here, the optimality equivalence means that the optimal solution of the fitness maximization problem corresponds to a minimum attribute reduction. Unfortunately, the existing fitness functions do not well meet the above mentioned requirements and consequently affect the performance of the related algorithms [19].

In this paper, an applicable fitness function was proposed. Compared with the existing fitness functions as mentioned earlier, it not only takes into account less factors but also overcomes the drawback. The experimental results show that, for each of the two tested algorithms, the use of the proposed fitness function has a higher probability to find a minimum reduction than the use of the function proposed in [19].

The rest of the paper is organized as follows.  Section 2 presents some concepts about minimum attribute reduction and reviewed and analysed a fitness function proposed in [19]. In Section 3, a new fitness function and properties are presented. In Section 4, the results of experiments and comparison analysis are given. Finally, Section 5 concludes the paper.

## 2. Basic Notions and Related Works

In this section, we will review some basic notions in the theory of rough sets which are necessary for the description of the minimum attribute reduction problem.

A decision table can be represented as *S* = {*U*, *A*, *V*, *f*}, where *U* = {*x*_1_, *x*_2_,…, *x*_*n*_} is a nonempty finite set of objects, *A* = *C* ∪ *D*, where *C* is a set of condition attributes and *D* is a decision attribute set, *V* is the domain of attributes belonging to *A*, and *f* : *U* × *A* → *V* is a function assigning attribute values to objects in *U*.

With any *R*⊆*A*, there is an associated indiscernibility relation IND(*R*):

(1) $$\begin{array}{c} IND\left(R\right)=\left\{\left(x,y\right)|f\left(x,a\right)=f\left(y,a\right),\;\forall a\in R,x,y\in U\right\}. \end{array}$$

Let *X*⊆*U*; the *R*-lower approximation of *X* is defined as $\underline{R}X=\{x\in U|{\left[x\right]}_{R}\subseteq X\}$, where [*x*]_*R*_ denotes an equivalence class of IND(*R*) determined by object *x*. The notation POS_*R*_(*D*) refers to the *R*-positive region given by ${POS}_{R}\left(D\right)=\cup_{X\in U/IND(D)}\underline{R}X$. The *R*-approximation quality with respect to decisions attribute set *D* is defined as *γ*_*R*_ = |POS_*R*_(*D*)|/|*U*|.

For decision table *S* = {*U*, *A*, *V*, *f*}, it may have many attribute reductions; the set of reductions is defined as

(2) Red=R⊆C ∣ POSRD=POSCD,  ∀B⊂R,POSRD≠POSBD.

For attribute reduction, the minimal attribute reduction with minimal cardinality will be searched. The minimal attribute reduction problem can be formulated as the following nonlinearly combinational optimization problem:

(3) minR⊂C Rs.t. POSRD=POSCD,POSRD≠POSBD, ∀B⊂R.

Let Red_*m*_ be a set which was defined as follows:

(4) $$\begin{array}{c} {Red}_{m}=\left\{R\in Red\;|\;\forall B\in Red,\left|B\right|\geq \left|R\right|\right\}. \end{array}$$

By the definition of Red_*m*_, the following proposition is apparent.

**Proposition 1 .**

Let *R* ⊂ *C*; then *R* is the optimal solution of the optimization problem (3) if and only if *R* ∈ *Red*_*m*_.

According to Proposition 1, each element of Red_*m*_ corresponds to a minimal reduction. In order to solve problem (3), the most commonly used approach is to transform it into the following unconstrained maximization problem and to solve it by heuristic algorithms:

(5) $$\begin{array}{c} \underset{R\subset C}{max}\;F\left(R\right), \end{array}$$

where *F*(*R*) is a fitness function about attribute subset *R*.

For this optimization problem, the equivalence of optimality between the minimum attribute reduction problem (3) and the fitness maximization of the function *F*(*R*) must be guaranteed. Unfortunately, most of the functions do not satisfy the requirement in the literatures [19].

For instance, Ye et al. defined a fitness function in [19]. Let

(6) $$\begin{array}{c} IND\left(C\right)=\left\{{\left[x_{1}\right]}_{C},{\left[x_{2}\right]}_{C},\ldots ,{\left[x_{N}\right]}_{C}\right\}, \end{array}$$

where the cardinality of the equivalence classes [*x*_*i*_]_*C*_ meets |[*x*_*i*_]_*C*_ | ≤|[*x*_*i*+1_]_*C*_|, where |·| denotes the cardinality of a set. The function was defined as

(7) $$\begin{array}{c} F_{1}\left(R\right)=\frac{m-\left|R\right|}{m}+\frac{n}{\Delta }\gamma_{R}, \end{array}$$

where Δ = |[*x*_1_]_*C*_ | >0.

Let *R* ⊂ *C*, |*R* | = |*C* | −1, and |POS_*R*_(*D*)| = |POS_*C*_(*D*)|−1; then *F*_1_(*C*) = |POS_*C*_(*D*)|/Δ and *F*_1_(*R*) = 1/*m* + (|POS_*C*_(*D*)|−1)/Δ, and thus *F*_1_(*C*) − *F*_1_(*R*) = 1/Δ − 1/*m*. If 1/Δ = 1/*m*, then *F*_1_(*C*) − *F*_1_(*R*) = 0. It suggests that the fitness function cannot distinguish between the two. That is to say, the two are equivalent when running the algorithm. This drawback will reduce the algorithmic performance. Moreover, in practical applications, since contains classification quality *γ*_*R*_, the value of the function *F*_1_(*R*) is not an integer. In the search process, the calculation error will reduce the probability of searching a minimum reduction. At the end, the consistency of decision table must be determined before calculating the fitness value, which increases the computational complexity.

## 3. A New Function and Its Properties

In this section, we present a new fitness function. Firstly, the relevant definitions were introduced as follows.

**Definition 2 .**

Let *R*⊆*C*. If POS_*R*_(*D*) = POS_*C*_(*D*) holds, then *R* is said to be a consistent set of *C*. Let *CS* be a set which includes all the consistent sets of *C* [19]:

(8) $$\begin{array}{c} CS=\left\{R\;|\;{POS}_{R}\left(D\right)={POS}_{C}\left(D\right),R\subseteq C\right\}. \end{array}$$

By the definition of reduction, for any consistent set *R* ∈ *CS*, *R* either is a reduction itself or contains a reduction. If *R* is minimal, then *R* is a reduction of *C*. It is very obvious that Red⊆*CS*. A nonlinearly constrained combinatorial optimization problem correlated with *CS* is defined as

(9) $$\begin{array}{c} \underset{R\in CS}{min}\left|R\right|. \end{array}$$

Let *CS*_*m*_ be a set which is defined as follows:

(10) $$\begin{array}{c} CS_{m}=\left\{R\in CS:\forall B\in CS,\left|B\right|\geq \left|R\right|\right\}. \end{array}$$

By the definition of *CS*_*m*_, we know that *CS*_*m*_ is a subset of *CS* and the following proposition is apparent.

**Proposition 3 .**

∀*R* ∈ *CS*_*m*_, then *R* is a reduction.

**Proposition 4 .**

Let *R* ⊂ *C*; then *R* ∈ *CS*_*m*_ if and only if *R* is the optimal solution of the optimization problem (9).

By Proposition 4, *CS*_*m*_ is the solution set of the optimization problem (9). According to the previous definition, we present a new fitness function as follows: ∀*R*⊆*C*,

(11) $$\begin{array}{c} F\left(R\right)=m\left(\left|{POS}_{C}\left(D\right)\right|-\left|{POS}_{R}\left(D\right)\right|\right)+\left|R\right|, \end{array}$$

where *m* = |*C*|.

**Proposition 5 .**

Let *P* ⊂ *C*.

(1) If *P* ∈ *CS*, ∀*R* ∈ *CS*_min_, then *F*(*R*) ≤ *F*(*P*) ≤ *m*.

(2) If *P* ∉ *CS*, then *F*(*P*) > *m*.

In particular, *F*(*P*) > *F*(*B*), ∀*B* ∈ *CS*.

**Proof**

If *P* ∈ *CS*, ∀*R* ∈ *CS*_min_, then |POS_*P*_(*D*)| = |POS_*R*_(*D*)| = |POS_*C*_(*D*)| and |*P* | ≤|*R* | ≤*m*. That is *F*(*R*) ≤ *F*(*P*) ≤ *m* according to the definition of the fitness function *F*(*R*). The (1) is hold. If *P* ∉ *CS*, then |POS_*C*_(*D*)|>|POS_*P*_(*D*)|, thus *F*(*P*) = *m*(|POS_*C*_(*D*)|−|POS_*P*_(*D*)|)+|*P* | >*m* + |*P* | >*m*.

According to Proposition 5, the codomain of the function *F*(*R*) can be divided into two disjoint sets [min*F*(*R*), *m*] and (*m*, max*F*(*R*)), where min*F*(*R*) and max*F*(*R*) are the minimum and maximum value of *F*(*R*), respectively. By (2), the defect of the function proposed in [19] was avoided. A minimum optimization problem is defined as follows by the definition of *F*(*R*):

(12) $$\begin{array}{c} \underset{R\subset C}{min}\;F\left(R\right). \end{array}$$

**Theorem 6 .**

Let *R* ⊂ *C*; then *R* ∈ *CS*_*m*_ if and only if *R* is a minimum attribute reduction; that is, *CS*_*m*_ = *Red*_*m*_.

**Proof**

*R* ∈ *CS*_*m*_; then *R* is a reduction by Proposition 3. If *R* is not a minimum attribute reduction, then there is a minimum attribute reduction *B* such that |*B* | <|*R*|.

On the other hand, since *B* is an attribute reduction, then *B* ∈ *CS*. By the definition of *CS*_*m*_ and *R* ∈ *CS*_*m*_, |*B* | ≥|*R*|, a contradiction and vice versa.

By Theorem 6, all the elements of *CS*_*m*_ are minimum attribute reduction; then according to Proposition 4, we can get that all the solutions of the optimization problem (9) are the minimum attribute reductions.

**Theorem 7 .**

∀*R* ⊂ *CS*_*m*_, then *R* is an optimal solution of the optimization problem (12). It is equivalent to the case where all the elements of *CS*_*m*_ are the optimal solutions of the optimization problem (12).

**Proof**

We use proof by contradiction. Assume that there is a *R* ∈ *CS*_*m*_ such that *R* is not an optimal solution of the optimization problem (12). Then ∃*B*⊆*C* such that *F*(*B*) < *F*(*R*). So

(13) 0>FB−FR=mPOSRD−POSBD+B−R.

Then, there are two cases of *B* that will be discussed in the following.

(1) If *B* ∈ *CS*, then POS_*B*_(*D*) = POS_*R*_(*D*) = POS_*C*_(*D*), so |POS_*B*_(*D*)|−|POS_*R*_(*D*)| = 0 and *F*(*B*) − *F*(*R*) = |*R* | −|*B*|. Since *R* ∈ *CS*_*m*_, thus |*R* | −|*B* | ≤0. Consequently

(14) $$\begin{array}{c} 0>F\left(B\right)-F\left(R\right)=\left|B\right|-\left|R\right|\geq 0, \end{array}$$

a contradiction.

(2) If *B* ∉ *CS*, then |POS_*B*_(*D*)|<|POS_*R*_(*D*)| = |POS_*C*_(*D*)|; that is, |POS_*R*_(*D*)|−|POS_*B*_(*D*)|≥1, and then

(15) $$\begin{array}{c} 0>F\left(B\right)-F\left(R\right)\geq m+\left|B\right|-\left|R\right|\geq \left|B\right|\geq 1, \end{array}$$

a contradiction.

From the above proof we know that all the elements of *CS*_*m*_ are the optimal solution of the optimization problem (12); that is to say, the minimum attribute reduction is the optimal solution of the optimization problem (12).

**Theorem 8 .**

Let *R* ⊂ *C*; if *R* is an optimal solution of the optimization problem (12), then *R* ∈ *CS*_*m*_.

**Proof**

(1)  *R* ∈ *CS*.

If *R* ∉ *CS*, then |POS_*R*_(*D*)|<|POS_*C*_(*D*)|. For all *B* ∈ *CS*, POS_*B*_(*D*) = POS_*C*_(*D*). Since *R* is an optimal solution of the optimization problem (12), then 0 ≥ *F*(*R*) − *F*(*B*), so

(16) 0≥FR−FB=mPOSBD−POSRD+R−B≥m+R−B≥1,

a contradiction, so *R* ∈ *CS*.

(2)  *R* ∈ *CS*_*m*_.

If *R* ∉ *CS*_*m*_, let *B* ∈ *CS*_*m*_. According to (1) we know *R* ∈ *CS*, so POS_*B*_(*D*) = POS_*R*_(*D*), and then

(17) $$\begin{array}{c} 0\geq F\left(R\right)-F\left(B\right)=\left|R\right|-\left|B\right|>0, \end{array}$$

a contradiction, so *R* ∈ *CS*_*m*_.

According to Theorem 8, the optimal solution of the optimization problem (12) is a minimum attribute reduction. Then, by Theorems 7 and 8, we can obtain the following theorem obviously.

**Theorem 9 .**

Let *R* ⊂ *C*; then *R* is an optimal solution of the optimization problem (12) if and only if *R* ∈ *CS*_*m*_.

By Theorem 9, we can get that the optimal solution of the optimization problem (12) is equivalent to the minimal attribute reduction. Then, according to Theorem 6, we can obtain the following theorem.

**Theorem 10 .**

Let *R* ⊂ *C*; then *R* is a minimum attribute reduction if and only if *R* is an optimal solution of the optimization problem (12).

By Theorem 10, the minimum value of the function proposed in this paper is equivalent to the minimal attribute reduction. We may get the minimum attribute reduction by searching the minimum value of function, and then stochastic category can be used.

## 4. Performance Comparison

In order to analyze and evaluate the effectiveness of the fitness function proposed in this paper, in the literature [19], an experiment was designed in the following way. Two existing minimum attribute reduction algorithms based on different types of stochastic optimization technique are used in the comparison. The first is the particle swarm optimization-based attribute reduction algorithm proposed in [20], denoted by PSO, and the other is a genetic algorithm based attribute reduction algorithm presented in [7, 8, 16], denoted by GA in short. In experiments, both algorithms were implemented and tested on a number of datasets using two different fitness functions: one is proposed in [19] (*F*_1_ in short) and the new fitness function is proposed in this paper (*F* in short). Seven datasets were chosen from the UCI machine learning repository. Most of these datasets are commonly used for evaluating attribute reduction algorithms in the literature [7, 8, 11, 14, 16, 17, 19].

For each of the two fitness functions, both algorithms were run 50 times on each of the datasets with the same setting of the parameters involved. For each run, three values need to be recorded: the first is the length of the output, the second is the output which corresponds to a reduction or not, and the last is run time. If an output is a reduction, then the output is said to be a normal output; otherwise, it is an unsuccessful output. If the length of the normal output is minimal, then the output is said to be a successful output. Let STL denote the length of the successful output, AVL denote the average length, and AVT denote the average run time during the 50 runs. The ratios of successful and normal outputs are denoted, respectively, by *s*_1_ and *s*_2_.

A PC running Windows 7 (32-bit) with 2.1 × 2 GHz CPU and 2 GB of main memory was used to run both algorithms. Both algorithms were programed by the MATLAB. Parameter settings for both algorithms were shown in Table 1. In Table 1, *p*-size refers to the size of population (in GA) or particle swarm (in PSO), *I*_max_ is the maximum allowed number of iterations (or generations), *V*_max_ is the upper bound on velocity needed in the PSO algorithm, *m*_*p*_ and *c*_*p*_ are the probabilities of mutation and crossover in GA, and *c*_1_ and *c*_2_ are the learning coefficients in PSO. Tables 2 and 3 present the main performance of PSO and GA using each of the two fitness functions.

From Table 2, for the algorithm PSO, on the index of STL, both fitness functions have the same value. It means that both fitness functions can output the minimum reduction. On the index of AVL, the value of *F* is not more than *F*_1_'s except for the last date. It reflects that the output of *F* is more focused on STL. The same conclusion can be arrived at from the index *s*_1_. It means that it has a higher probability to find a minimum reduction by using the proposed fitness function. For AVT, all the values of *F* are less than the values of *F*_1_. Therefore, the experimental data show that the efficiency of the proposed fitness function is better than *F*_1_ by using the algorithm PSO. The same conclusion can be obtained from Table 3 by using the algorithm GA.

The above two experiments show that the proposed fitness function is more adequate than the other fitness functions on the datasets.

## 5. Conclusions

In this paper, in order to overcome the drawback of the existing fitness functions for the problem of minimum attribute reduction, we discussed the fitness function and a simpler fitness function was proposed in this paper. Theoretical analysis and experimental results show that it can ensure the optimality equivalence and is more adequate than the existing fitness function.

## Figures and Tables

**Table 1:** Parameter settings for both algorithms.

Algorithms	*p*-size	*V*_max⁡_	*I*_max⁡_	*c*_1_	*c*_2_	*m*_*p*_	*c*_*p*_
PSO	20	7	100	2.0	2.0	—	—
Gen	50	—	100	—	—	0.4	0.6

**Table 2:** Performance of PSO using different fitness functions.

Dataset	*F*_1_				*F*			
	STL	AVL	*s*_1_/*s*_2_	AVT	STL	AVL	*s*_1_/*s*_2_	AVT
blance	4	4	1/1	35.392	4	4	1/1	26.467
spect	16	16	1/1	7.471	16	16	1/1	6.921
Lung-cancer	3	4.02	0.08/1	2.628	3	**3.92**	**0.22**/1	1.096
Sponge	8	8.4	0.61/0.98	5.327	8	**8.02**	**0.79**/1	4.217
Vote	8	8.2	0.8/1	7.419	8	**8.13**	**0.87**/1	5.522
Zoo	5	5.05	0.96/1	5.092	5	**5**	**1**/1	4.275
Soybean-small	2	**2.32**	**0.78**/1	2.091	2	2.38	0.72/1	1.162

**Table 3:** Performance of GA using different fitness functions.

Dataset	*F*_1_				*F*			
	STL	AVL	*s*_1_/*s*_2_	AVT	STL	AVL	*s*_1_/*s*_2_	AVT
blance	4	4	1/1	38.501	4	4	1/1	30.359
spect	16	16	1/1	8.021	16	16	1/1	7.856
Lung-cancer	3	4.21	0.07/0.98	2.983	3	**4.03**	**0.2**/**1**	1.123
Sponge	8	8.58	0.59/0.96	6.306	8	**8.17**	**0.77**/0.98	4.311
Vote	8	8.31	0.78/1	8.762	8	**8.22**	**0.82**/1	5.762
Zoo	5	5.11	0.94/1	6.243	5	**5**	**1**/1	4.52
Soybean-small	2	**2.43**	**0.76**/1	2.227	2	2.51	0.71/1	1.175
